# The transcriptional response of *Arcobacter butzleri* to cold shock

**DOI:** 10.1002/2211-5463.12959

**Published:** 2020-09-19

**Authors:** Xiaochen Zhang, Yulan Su, Thomas Alter, Greta Gölz

**Affiliations:** ^1^ Institute of Food Safety and Food Hygiene Freie Universität Berlin Berlin Germany

**Keywords:** *A. butzleri*, cold adaptation, cold shock‐related genes, temporal transcriptional expression

## Abstract

*Arcobacter* (*A*.) *butzleri* is an emerging zoonotic pathogen associated with gastrointestinal diseases, such as abdominal cramps and diarrhea, and is widely detected in animals, showing a high prevalence in poultry and seafood. The survival and adaptation of *A. butzleri* to cold temperatures remains poorly studied, although it might be of interest for food safety considerations. To address this, growth patterns of eight *A. butzleri* isolates were determined at 8 °C for 28 days. *A. butzleri* isolates showed strain‐dependent behavior: six isolates were unculturable after day 18, one exhibited declining but detectable cell counts until day 28 and one grew to the stationary phase level. Out of 13 *A. butzleri* cold shock‐related genes homologous to *Escherichia coli*, 10 were up‐regulated in response to a temperature downshift to 8 °C, as demonstrated by reverse transcription‐quantitative PCR. Additionally, we compared these data with the cold‐shock response in *E. coli*. Overall, we provide a deeper insight into the environmental adaptation capacities of *A. butzleri*, which we find shares similarities with the *E. coli* cold‐shock response.

## Graphical abstract

*Arcobacter butzleri* isolates showed strain‐dependent growth patterns at 8 °C. In addition, 13 genes homologous to cold shock‐related genes of *Escherichia coli* were identified in the genome of the *A. butzleri* reference strain, and similar expression patterns of these genes were observed by reverse transcription‐quantitative PCR in the early phase after cold shock.

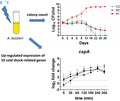

**Abbreviations**
BHI: brain heart infusion
CFU: colony‐forming unit
RT‐qPCR: reverse transcription‐quantitative PCR

*Arcobacter* spp. is an emerging zoonotic pathogen associated with gastrointestinal diseases such as abdominal cramps and diarrhea [1, 2, 3]. Among the 29 species within the genus of *Arcobacter* described so far [4], *A*. *butzleri* is the most widely detected species in animals, environment and foods, with high prevalences in poultry meat and seafood samples [5, 6, 7, 8, 9, 10]. Although the good environmental adaptability of *A. butzleri* is well known, the survival and adaptation of *A. butzleri* to cold remains poorly studied so far, although it might be of interest for food safety considerations. *A. butzleri* has an optimal growth temperature of 30 °C under both aerobic and microaerobic conditions but is likely to be exposed to a wide temperature range during its transmission cycle. The minimum growth temperature of *A. butzleri* was initially reported to be 15 °C [11]. Later, some studies reported lower limits as 10 °C; for example, in Ellinghausen McCullough Johnson Harris medium [12] and in chicken meat juice, as well as in Brain Heart Infusion (BHI) broth [13]. Van Driessche and Houf [14] investigated the survival capability of *A. butzleri* in water with/without organic material at minimum 4 °C and reported that all tested *A. butzleri* strains survived for at least 203 days when organic material is present in the water, indicating the best cold adaptation among all human‐related *Arcobacter* species.

With the complete genome of the *A. butzleri* RM4018 sequenced in 2007 [15], the understanding of adaptation to environmental stress could be improved. To date, no data describing the cold‐shock response of *Arcobacter* spp. have been reported. Because previous phenotypic studies suggest that *A. butzleri* could adapt to cold temperatures, the presence of cold‐related genes is presumed.

The function of the cold‐related proteins is best studied in *Escherichia coli*. Jones *et al*. [16] first reported that, after a cold shock from 37 °C to 10 °C, the transcription and translation processes for a majority of genes slow down or come to an almost complete stop, whereas a set of cold‐shock proteins is preferentially and transiently expressed [17, 18, 19, 20]. These proteins were classified in two groups according to their expression pattern [21]. The class I proteins include: CspA (the major cold‐shock protein of *E. coli,* encoded by *cspA*), DeaD (reassigned as CsdA, a DEAD‐box protein, encoded by *deaD*), NusA (the transcription termination/antitermination protein, encoded by *nusA*), RbfA (the ribosome binding factor A, encoded by *rbfA*) and RNase R (encoded by *rnr*). The induction of the expression level of these proteins varies but is more pronounced compared to class II proteins that include PNPase (the polynucleotide phosphorylase, encoded by *pnp*), IF‐2 (the translation initiation factors IF2, encoded by *infB*), GyrA (the DNA gyrase subunit A, encoded by *gyrA*), RecA (a DNA‐dependent ATPase, encoded by *recA*), DnaA (the replication initiator protein, encoded by *dnaA*), trigger factor (encoded by *tig*) and pyruvate dehydrogenase (encoded by *aceE* and *aceF*). All of the above‐mentioned genes have been reviewed by Gualerzi *et al*. [18] and Barria *et al*. [20].

Because little is known about the adaption of *A. butzleri* to cold, the growth capabilities of eight *A. butzleri* isolates derived from human, mussels and chicken were investigated at 8 °C and expression at the transcriptional level of the previously described 13 cold‐related genes was studied in three *A. butzleri* strains showing different growth and survival capability after a temperature down shift from 28 °C to 8 °C.

## Materials and methods

### Bacterial strains and growth conditions

The *A. butzleri* strains used in this study were previously described [10, 22]. All isolates from human feces (H1, H2 and H3), mussels (M3 and M4) and chicken meat (C1, C2 and C3) were routinely grown in Brucella Broth (BD, Heidelberg, Germany) or on Mueller‐Hinton blood agar (Oxoid, Wesel, Germany). The working cultures were maintained under aerobic atmosphere at optimal (28 °C) or cold (8 °C) temperatures.

### Growth curve

Briefly, all *A. butzleri* strains were recovered on Mueller‐Hinton blood agars under microaerobic conditions (6% O_2_, 10% CO_2_) for 72 h at 28 °C. For each strain, 5 mL of Brucella Broth was inoculated and incubated overnight (18 h) under microaerobic conditions at 28 °C to reach approximately 8 log_10_ colony‐forming units (CFU)·mL^−1^. These pre‐cultures were subsequently diluted 1:10 000 in 5 mL of pre‐cooled (8 °C) or pre‐warmed (28 °C) Brucella Broth, respectively. The cultures were incubated at the respective temperatures under aerobic conditions and the bacterial load was determined over 28 days. At each indicated time point, serial dilutions of the cultures were plated on Mueller‐Hinton blood agar plates and incubated for 48 h at 28 °C under microaerobic conditions before enumeration.

### RNA extraction

To screen the transcriptional expression pattern of the presumed cold‐related genes in *A. butzleri* at a very early stage after the sudden temperature downshift, the RNA of *A. butzleri* isolates from different growth modes at cold temperatures (H2 and C2) and the reference strain (H1/*A. butzleri* RM4018) were analyzed. Briefly, pre‐cultures were prepared as described above but in a volume of 300 mL of fresh Brucella Broth. After overnight incubation, the cultures reached the post‐logarithmic phase and were harvested by centrifugation (5 min at 7500 ***g***) at room temperature and resuspended in 200 mL of fresh pre‐cooled (8 °C) Brucella Broth. The master culture was split equally into eight working cultures. All working cultures were incubated at 8 °C except the reference sample (0 min), which was further processed immediately. Samples were collected at 5, 30, 60, 120, 180, 240 and 360 min after the temperature downshift. Bacteria from each sample were harvested by centrifugation (5 min at 7500 ***g***) at 8 °C, the pellet resuspended in 1 mL RNA Protect (Qiagen, Hilden, Germany) and stored at −80 °C until further processing. RNAs were isolated by peqGOLD Bacteria RNA Kit (VWR, Radnor, PA, USA) in accordance with the manufacturer's instructions (Thermo Scientific, Waltham, MA, USA). Afterwards, the RNA was quantified by a NanoDrop 2000 (Thermo Scientific) and subsequently subjected to DNase treatment. Briefly, a total volume of 40 µL containing 750 ng of RNA, 4 U of DNase I, 1 × Reaction Buffer with MgCl_2_, 40 U of RiboLock (all from Thermo Scientific) and nuclease‐free water was incubated at 37 °C for 15 min. The DNase digestion reaction was terminated by addition of 4.5 mm EDTA (Thermo Scientific) and incubation at 65 °C for 10 min. All samples were placed on ice for further steps afterwards.

### cDNA transcription

The cDNA of the RNA samples were synthesized using Maxima H Minus First Strand cDNA Synthesis Kit (Thermo Scientific) in accordance with the manufacturer's instructions. Briefly, the cDNA synthesis mix contained 220 ng of RNA, 0.5 mm dNTP Mix and 5 µm Random Hexamer Primer in a total volume of 15 µL. The mix was pre‐heated to 65 °C for 5 min, before the addition of 4 μL of 5 × RT Buffer and 1 μL of Maxima H Minus Enzyme Mix, and incubated at 25 °C (10 min) followed by 15 min incubation at 50 °C. The reaction was terminated by 5 min incubation at 85 °C. To confirm the efficiency of the DNase digestion, a control without addition of the reverse transcription enzyme of each sample was carried out in parallel. The cDNA was diluted in water at a ratio of 1:10 for reverse transcription‐quantitative PCR (RT‐qPCR).

### Reverse transcription‐quantitative PCR

The gene expression profile was analyzed by RT‐qPCR. Primers used in this assay were designed using primer3 (http://frodo.wi.mit.edu) and are listed in Table 1. The RT‐qPCR was carried out using SsoFastTM EvaGreen Supermix® (Bio‐Rad, Munich, Germany) and the CFX Connect Real‐Time System (Bio‐Rad) in accordance with the manufacturer's instructions. Briefly, 7.5 μL of SsoFast EvaGreen Supermix, 36 nmol of each forward and reverse primer, and 1 μL of cDNA was mixed in a total volume of 15 µL. The RT‐qPCR was started by an initial preheating step for 30 s at 95 °C, followed by 40 cycles of 5 s of denaturation at 95 °C and 5 s of annealing (corresponding annealing temperatures for each gene is shown in Table 1). Here, *rpoA* was used as housekeeping gene for normalization of expression. Fold changes were calculated according to the ΔΔ*C*_T_ method [23] and genes with an expression ratio ≥ 2.0 were considered to be up‐regulated and those with an expression ratio ≤ 0.5 were considered to be down‐regulated [24]. The expression of each gene was analyzed using three independent cDNA samples with two technical replicates in each run.

**Table 1:** List of primers and annealing temperatures used in the RT‐qPCR assays.

Primer ID	Sequence (5'‐ to 3')	Target gene	Amplicon	Annealing	Strain
Abu_1472 f	AGC CTG AAT CAC TTG GAG CT	*aceE*	164	60 °C	C2, H1, H2
Abu_1472 r	AAC TTC CCA TCC AGC TCC TC				
Abu_1473 f	GGA GTT GCT GTT GAT ACA CCA	*aceF*	200	60 °C	C2, H1, H2
Abu_1473 r	GGC GTG AAG TAT GTT CCA CC				
Abu_0042 f	TGG ACA AGC GCA TAC AGG TA	*deaD*	195	60 °C	C2, H1
Abu_0042 r	GCT TGT CCA CCG TAA ACA GT				
deaD 1‐F	ACAGCAGCTTTTGGACTTCC	*deaD*	167	55 °C	H2
deaD 1‐R	GCTTGACCACCGTAAACAGT				
Abu_0001 f	CGT TGT GGG ACC ATC AAA CC	*dnaA*	108	60 °C	C2, H1, H2
Abu_0001 r	CCA AGT CCC GTT CCA CCA TA				
Abu_2043 f	TGA TAC TCC AGG TCA CGC AG	*infB*	91	60 °C	C2, H1
Abu_2043 r	GTC ATC AGC AGC AAC AAC GA				
infB 1‐F	AGGATTTAATGTACGACCAA	*infB*	129	52 °C	H2
infB 1‐R	CTCATCATCCCTGAAAGTG				
Abu_1183 f	TGC TGG ACC TAA AGA TGG ACA	*pnp*	108	60 °C	C2, H1, H2
Abu_1183 r	GCT TCA CCA ACG CTA AAT CCT				
Abu_2241 f	TAA ATG CAC AAA CGC CAC CA	*recA*	131	60 °C	C2, H1, H2
Abu_2241 r	AGC TTT AGG TGT TGG AGG ACT				
Abu_1702 f	ACC GTC AAA TGC AAC ACC AT	*tig*	112	60 °C	C2, H1, H2
Abu_1702 r	TGC TCA ATC ATC TGC ACC AT				
Abu_0246 f	ACC TTT TAT TTC GCC AAT TTC GA	*nusA*	176	55 °C	C2, H2
Abu_0246 r	TGG GAA GAA ATG CTG CAA CA				
nusA 1‐F	TGG AGA TGT TGT AAA AGC TGT TG	*nusA*	240	55 °C	H1
nus‐A 1‐R	ACT CCA ACA ACT GCA CCA AT				
rbfA 1‐R	CGC AAT AGT GTA TTT TGA TGG T	*rbfA*	127	55 °C	C2, H1, H2
rbfA 1‐F	ACA TTT ATA CCA ACT TGT ACT TGC				
cspA 1‐R	TGG CAA ATC AAA ATA TCG GAA CA	*cspA*	197	55 °C	C2, H1, H2
cspA 1‐F	TGC TTG AGG ACC TTT ATC ATT				
rnr 1‐F	AGG GCA TTT TGG TTT AGG ATT CT	*rnr*	205	55 °C	C2, H1, H2
rnr 1‐R	AGC CCA TCT TGC GTA TTT TCT				
gyrA 1‐F	TGG ACG TGC ATT ACC TGA TG	*gyrA*	192	55 °C	C2, H1
gyrA 1‐R	TGT GCC ATT CTT ACA AGT GCA				
gyrA 2‐F	TTGGACGTGCGTTACCTGAT	*gyrA*	193	55 °C	H2
gyrA 2‐R	TGTGCCATTCTTACAAGTGCA				
AB_rpoA_ex F	TAGCCCACCCTTTGAGAAGA	*rpoA*	50	60 °C, 55 °C, or 52 °C^a^	C2, H1, H2
AB_rpoA_ex R	CGCACAACCAACTGATGAAC				

^a^ Depending on the corresponding protocol used.

### Statistical analysis

Temporal expression patterns of the selected *A. butzleri* genes were analyzed and compared by *K*‐means clustering using genesis, version 1.8.1 (TU Graz, Graz, Austria) with three clusters, a maximum of 10 000 iterations and five runs.

The difference in temporal expression pattern among three *A. butzleri* isolates was analyzed by two‐way analysis of variance using prism, version 6.00 (GraphPad Software Inc., La Jolla, CA, USA).

## Results and Discussion

### Growth curves of *A. butzleri* isolates

**Fig. 1:**
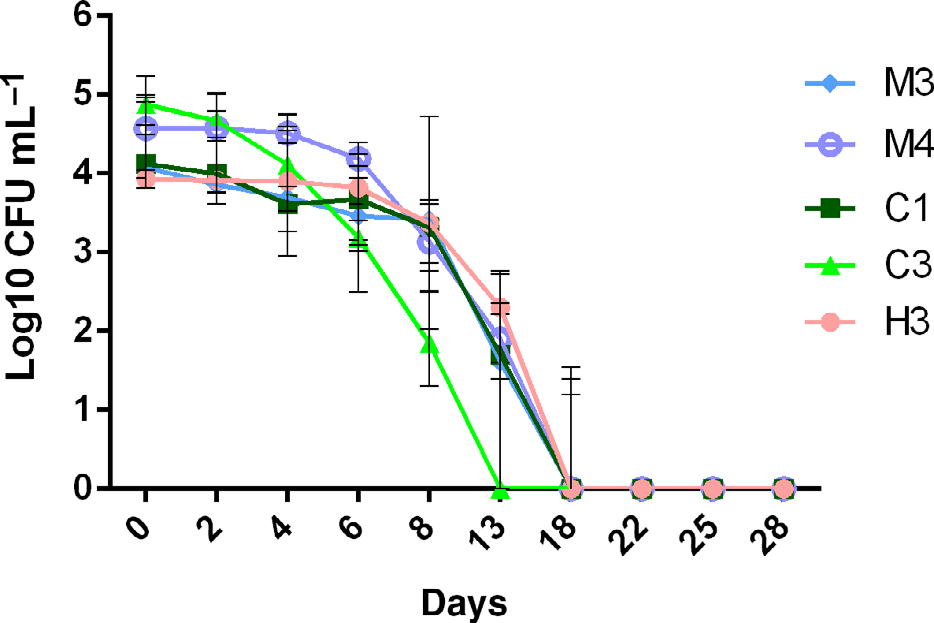
Growth curve of five *A. butzleri* isolates with no growth capability at 8 °C, shown as the median ± interquartile range (*n* = 3).

**Fig. 2:**
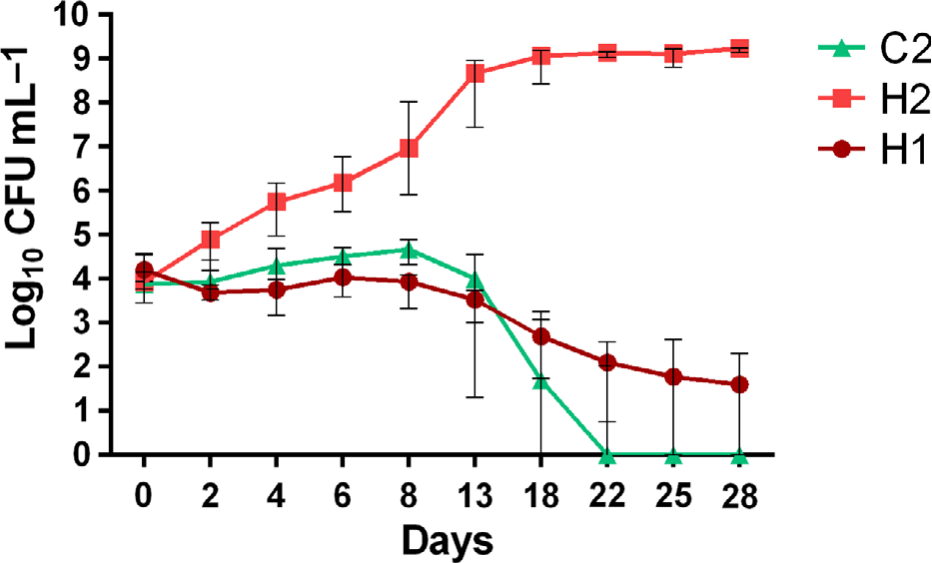
Growth curve of three *A. butzleri* isolates at 8 °C, shown as the median ± interquartile range (*n* = 4).

Because *A. butzleri* is highly prevalent in chicken meat and seafood products, *A. butzleri* strains isolated from these matrices, as well as from a human stool specimen, were included in our cold adaptation study. The growth of eight *A. butzleri* isolates from human (H), mussels (M) and chicken (C) was determined at 28 °C and 8 °C for 28 days or until undetectable on Mueller‐Hinton blood agar plates. No difference in the growth capabilities of all eight strains could be determined during incubation at 28 °C under aerobic conditions (data not shown). For six of the eight investigated isolates (75%), stable CFU counts of approx. 4 log_10_ CFU·mL^−1^ were determined until 8 days after incubation at 8 °C, followed by declining CFU counts until 22 days, whereas, afterwards, no colony counts could be determined (Fig. 1). This behavior is represented by strain C2 in Fig. 2. For the reference strain H1 (*A. butzleri* RM4018), a declining tendency of cell counts by 2 log_10_ level was also determined, although colonies were still detectable on Mueller‐Hinton blood agar plates at day 28 (Fig. 2). Overall, we determined only survival for strain H1 at 8 °C; however, in two of eight replicates, we observed slightly growing capabilities of this strain. By contrast, the strain H2 was able to grow at 8 °C, reaching the stationary phase with 9 log_10_ CFU·mL^−1^ around 13 days in all replicates (Fig. 2). No correlation between the strain origin and the growth behavior at 8 °C was observed, which is comparable with results of the previous study by Van Driessche and Houf [14].

To the best of our knowledge, 8 °C was the lowest temperature to ever been reported for the observation of continued growing up to 5 log_10_ level of *A. butzleri* under aerobic conditions in media. Consistent with a previous study, a low temperature resulted in delayed growth in any medium [13]. In a study by Kjeldgaard *et al*. [13], *A. butzleri* strain ATCC 49616 (H1 in the present study) was able to grow at 10 °C in BHI broth and chicken meat juice and could survive in both media when stored at 5 °C. A recent study by Šilha *et al*. [25] observed non‐significant multiplication with gradually decreasing viability until 14 days for *A. butzleri* CCUG 30484 (H1 in the present study) at 5 °C in BHI culture.

In previous reports, *A. butzleri* isolates were inoculated in different food matrices to investigate their growth and survival capability at different refrigeration temperatures. In ultrahigh‐temperature‐treated, pasteurized and raw milk, *A. butzleri* could remain viable at both 4 °C and 10 °C until 6 days [26]. *A. butzleri* RM4018 count remained stable during 5 days of storage at 6 °C in artisanal ricotta cheese, although it decreased by 2.5 log_10_ level during 22 days storage in industrial ricotta cheese. However, the *A. butzleri* count increased by up to 8 log_10_ CFU·g^−1^ in industrial cheese sample at 7 days and remained stable until the end of the shelf life (22 days) when stored at 12 °C [27]. These results confirmed the hypothesis that the critical growing temperature of the strain *A. butzleri* RM4018 was between 6 and 12 °C at least in cheese media. Other studies also demonstrate the growing ability of *A. butzleri* at even lower temperatures, although only over a short time period. Van Driessche and Houf [14] reported slightly increasing cell counts of some *A. butzleri* strains within 7 days at 4 °C and 7 °C in pure water before the counts declined. Likewise, Badilla‐Ramirez *et al*. [28] tested the survival rate of *A. butzleri* on chicken legs. Their results showed that *A. butzleri* had a short growing capacity until 3 days at 4 °C and 10 °C followed by a declining tendency afterwards.

Taken together, we suggest that, even though cell counts of the majority of *A. butzleri* isolates start to decline after 8 days of storage at 8 °C, some strains were able to maintain a stable cell count or could even grow at this temperature.

### The expression profiles of selected *A. butzleri* genes after cold shock at 8 °C

**Fig. 3:**
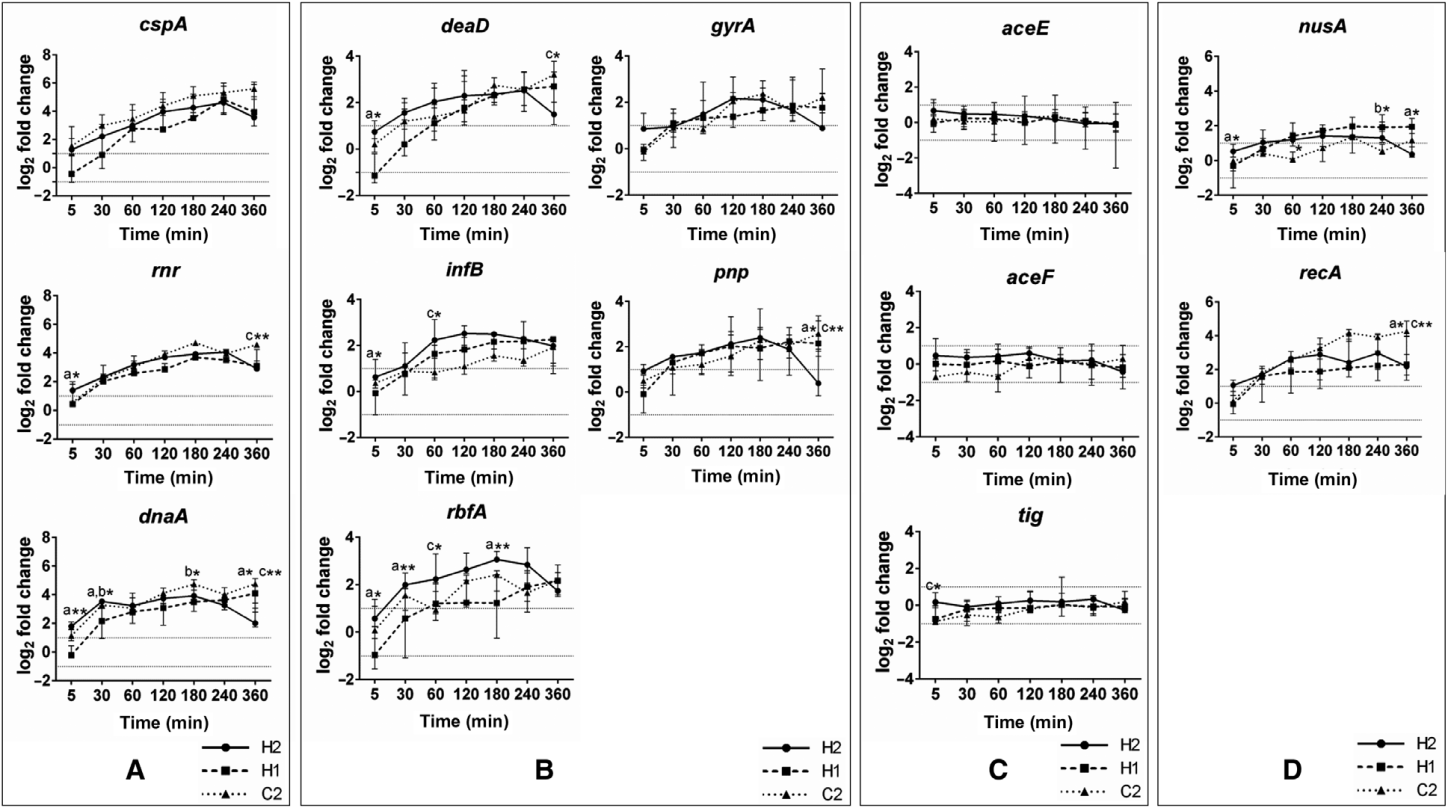
Temporal expression pattern of 13 cold‐related genes in three *A. butzleri* isolates after a temperature downshift to 8 °C. The expression level was analyzed by RT‐qPCR and is shown as the median ± interquartile range of log_2_ fold changes (*n* = 3). The dotted lines show the threshold (−1 to 1) for the relevant up‐ and down‐regulation. Similar expression pattern were analyzed by *K*‐means clustering. Cluster I includes genes with high up‐regulated expression (A), cluster II includes those with moderate up‐regulated expression (B) and cluster III includes those with non‐regulated expression (C) in all three *A. butzleri* strains. (D) Genes belonging to different clusters in strain H1, H2 and C2. Statistical significant difference between strain H1 and H2 (a), H1 and C2 (b) or H2 and C2 (c): *P < 0.05, **P < 0.01 (analyzed by two‐way analysis of variance).

Even though several studies have described the prolonged survival or growth at lower temperatures for *A. butzleri*, knowledge of the cellular processes involved in adaptation to cold temperature for this species is still scarce. In general, one of the most important cold‐shock response mechanisms of bacterial species is known to sustain the translational processes, which are strongly impeded at low temperatures [29]. For several proteins involved in the cold stress response of *E. coli*, homologous genes could be determined in the genome of *A. butzleri*, including the genes encoding for RNA modifying proteins (*cspA*, *rnr*, *deaD* and *pnp*), translational regulators (*infB*, *rbfA*), chromosome modulators (*gyrA, recA*), transcriptional regulators (*nusA, dnaA*), a protein chaperon (*tig*) and metabolic enzymes (*aceE*, *aceF*). The expression profiles of these putative cold‐related genes were investigated by RT‐qPCR after a temperature down shift to 8 °C under aerobic conditions for the *A. butzleri* isolates H2 and C2 (representing different growth tendencies at cold temperatures) and the reference strain H1. Temporal expression patterns of the genes were analyzed and compared by *K*‐means clustering. As shown in Fig. 3, the expression level of 10 of the 13 investigated genes (77%) was up‐regulated in response to the cold shock, and the genes were grouped into the high up‐regulated cluster I (> 4.5 log_2_ fold change) or the moderate up‐regulated cluster II (1.5–3 log_2_ fold change). For all three strains, the expression patterns of *cspA*, *rnr* and *dnaA* were grouped in the high up‐regulated cluster I (Fig. 3A), whereas *deaD*, *gyrA*, *infB*, *pnp* and *rbfA* were grouped in the moderate up‐regulated cluster II (Fig. 3B). Cluster III comprises the genes *aceE*, *aceF* and *tig,* for which the expression levels were non‐regulated within the first 6 h after cold shock in all three isolates (Fig. 3C). By contrast to the above mentioned similarities in the gene expression pattern, differences could be observed for the expression pattern of *recA* and *nusA* in the three investigated *A. butzleri* strains (Fig. 3D). For the strains H1 and H2, the expression pattern of both genes were grouped into the moderately up‐regulated cluster II, whereas *recA* was included in the high up‐regulated cluster I and *nusA* was grouped in the non‐regulated cluster III for the strain C2 (Fig. 3D).

To the best of our knowledge, no data describing the cold‐shock response of *Arcobacter* spp. have been published so far; therefore, we compared our data with *E. coli,* for which the cold‐shock response has been intensively studied [19]. Recently, a comprehensive study by Zhang *et al*. [30] demonstrated a two‐member mRNA surveillance system composed of the exonuclease RNase R and the RNA chaperon CspA, enabling the recovery of translation during cold acclimation in *E. coli*. Enhanced mRNA levels for *cspA* and *rnr* were also determined for all three *A. butzleri* isolates, suggesting utilization of a similar control mechanism. However, similar to *E. coli*, the mRNA level reached a maximum at 4 h after cold shock and then decreased in strains H1 and H2, whereas it did not decline in strain C2 until the end of the assay.

Furthermore, we determined a similar tendency of gene expression of the other 11 corresponding genes investigated in the present study compared to *E. coli* [30]. Enhanced expression level for *deaD* and *pnp*, both encoding for components of the RNA degradosome [31], have also been determined for all three *A. butzleri* isolates. However, for *E. coli*, a higher expression level for *deaD* could be determined compared to the mRNA level of *deaD* in *A. butzleri*.

Similar to our results for *A. butzleri*, no changes in mRNA level of the genes *aceE*, *aceF* (both involved in the pyruvate metabolism) and *tig* (a protein chaperon, active at the later stage of adaptation to cold temperatures [32]) were reported for *E. coli* within the investigated 6 h after cold shock [30].

The expression level of *dnaA*, the chromosomal replication initiator protein, was increased in all three *A. butzleri* strains, as well as in *E. coli*. However, for *A. butzleri*, the expression pattern was grouped into the highly regulated cluster, whereas its expression pattern in *E. coli* is only moderately enhanced. However, the precise role of enhanced *dnaA* mRNA/protein level has not yet been clarified in detail.

In *E. coli*, the genes *nusA‐infB‐rbfA* are located in an operon and mRNA levels for all three genes increased after cold shock [33]. In *A. butzleri*, only *infB* and *rbfA* (Abu_2043 and Abu_2044) are located in the direct neighborhood, whereas *nusA* (Abu_0246) is located on a different region on the chromosome. Moderate enhanced expression levels of *rbfA* (encoding a protein rendering the ribosomes to translate non‐cold‐shock mRNAs under cold conditions [31]) and *infB* (encoding the translation initiation factor IF2) were determined for all three *A. butzleri* isolates, which is also in concordance to data reported for *E. coli* [30]. By contrast, differences in the *nusA* expression pattern between the three *A. butzleri* strains were determined. The protein NusA is a transcription factor functionally involved in the regulation of pausing of the RNA polymerase during RNA chain elongation, as well as in the termination of transcription [34, 35]. Comparable to *E. coli*, the expression level of *nusA* was moderately increased in the strains H1 and H2, whereas it was not increased in the strain C2.

Furthermore, the expression of *recA* is only moderately induced in the two *A. butzleri* strains H1 and H2, as well as in *E. coli* [30], although it is highly induced in the *A. butzleri* strain C2. RecA is the major DNA recombinase involved in homologues recombination and DNA repair [36]. Another protein involved in the chromosomal structure is the DNA gyrase alpha subunit (GyrA), which regulates chromosomal DNA supercoiling upon cold shock and also provides a level of DNA supercoiling and favors its efficient transcription [32]. The expression of *gyrA* is regulated by CspA [37] and, consistently, we determined moderately enhanced expression level for *gyrA* in all three *A. butzleri* isolates.

Taken together, we have determined several similarities in the cold‐shock response of *A. butzleri* with *E. coli*. However, additional studies are required to clarify whether and how differences in the expression levels of *nusA* and *recA* between the three *A. butzleri* might be responsible for the different growth behaviors determined at low temperatures under aerobic conditions.

## Conclusions

Overall, our data indicate that most of the analysed *A. butzleri* isolates from human, mussel and chicken were not able to grow at 8 °C under aerobic conditions. However, one *A. butzleri* isolate was able to grow to the stationary phase until the end of the assay.

Furthermore, the presence of 13 homologues genes encoding cold shock‐related proteins in *E. coli* was determined in the genome of *A. butzleri*, including *cspA*, *rnr*, *deaD*, *pnp*, *infB*, *rbfA*, *gyrA*, *recA*, *nusA*, *dnaA*, *tig*, *aceE* and *aceF*. The temporal transcriptional expression profiles of these genes in the early cold stress response of *A. butzleri* are reported for the first time and showed high similarities compared to *E. coli*. These data provide deeper insight into the environmental adaptation capacities of *A. butzleri*.

## Conflict of interests

The authors declare that they have no conflicts of interest.

## Author contributions

GG and TA supervised the study. XZ and GG designed the experiments. XZ and YS performed the experiments. XZ and GG analyzed data. XZ wrote the manuscript. XZ, GG, YS and TA made manuscript revisions.

## Data Availability

The raw data of this study are available from the corresponding author on reasonable request.
